# Tezosentan-induced attenuation of lung injury in endotoxemic sheep is associated with reduced activation of protein kinase C

**DOI:** 10.1186/cc3497

**Published:** 2005-03-14

**Authors:** Vladimir Kuklin, Mikhail Kirov, Mikhail Sovershaev, Thomas Andreasen, Ole C Ingebretsen, Kirsti Ytrehus, Lars Bjertnaes

**Affiliations:** 1 Research Fellow, Department of Anesthesiology, Faculty of Medicine, University of Tromsø, Norway; 2 Research Fellow, Department of Physiology, Faculty of Medicine, University of Tromsø, Norway; 3 Departmental engineer, Department of Physiology, Faculty of Medicine, University of Tromsø, Norway; 4 Professor, Department of Clinical Chemistry, University Hospital of Tromsø, Norway; 5 Professor, Department of Physiology, Faculty of Medicine, University of Tromsø, Norway; 6 Professor, Chairman of the Department of Anesthesiology, Faculty of Medicine, University of Tromsø, Norway

## Abstract

**Introduction:**

Studies *in vitro *reveal that endothelin-1 (ET-1) activates the α isoform of protein kinase C (PKC-α) in cultures of endothelial cells, thereby deranging cellular integrity. Sepsis and endotoxemia are associated with increased plasma concentrations of ET-1 that induce acute lung injury (ALI). We recently reported that non-selective ET-1 receptor blockade attenuates ALI in sheep by reducing the endotoxin-induced increase in extravascular lung water index (EVLWI). The aim of this study was to find out whether this attenuation is associated with reduced translocation of PKC-α from the cytosolic to the membrane fraction of lung tissue homogenate.

**Methods:**

Seventeen awake, instrumented sheep were randomly assigned to a sham-operated group (n = 3), a lipopolysaccharide (LPS) group (n = 7) receiving an intravenous infusion of Escherichia coli 15 ng/kg per min for 24 hours, and a tezosentan group (n = 7) subjected to LPS and, from 4 hours, an intravenous injection of tezosentan 3 mg/kg followed by infusion at 1 mg/kg per hour for the reminder of the experiment. Pulmonary micro-occlusion pressure (Pmo), EVLWI, plasma concentrations of ET-1, tumor necrosis factor-a (TNF-a), and interleukin-8 (IL-8) were determined every 4 hours. Western blotting was used to assess PKC-α.

**Results:**

In non-treated sheep a positive correlation was found between the plasma concentration of ET-1 and Pmo in the late phase of endotoxemia (12 to 24 hours). A positive correlation was also noticed between Pmo and EVLWI in the LPS and the LPS plus tezosentan groups, although the latter was significantly reduced in comparison with LPS alone. In both endotoxemic groups, plasma concentrations of ET-1, TNF-α, and IL-8 increased. In the LPS group, the cytosolic fraction of PKC-α decreased by 75% whereas the membrane fraction increased by 40% in comparison with the sham-operated animals. Tezosentan completely prevented the changes in PKC-α in both the cytosolic and the membrane fractions, concomitantly causing a further increase in the plasma concentrations of ET-1, TNF-α, and IL-8.

**Conclusion:**

In endotoxemic sheep, ET-1 receptor blockade alleviates lung injury as assessed by a decrease in EVLWI paralleled by a reduction in Pmo and the prevention of activation of PKC-α.

## Introduction

Endothelin-1 (ET-1) has been identified as the most potent vasoconstrictor peptide known so far [1,2]. Locally produced ET-1 acts on three types of G-protein-coupled receptor: ET_A_, ET_B1_, and ET_B2 _[3]. The ET_A _and ET_B2 _receptors are expressed in vascular smooth muscle cells, whereas ET_B1 _is localized mainly in the endothelium. Binding of ET-1 to ET_A _and ET_B2 _leads to vascular constriction, whereas ET_B1 _induces relaxation by releasing nitric oxide and prostacyclin [4]. In the lowest concentration range, ET-1 mainly acts on the ET_B1 _receptor [5].

In sepsis, endotoxin and other microbial products that are released into the bloodstream trigger endothelial cells to the enhanced generation of ET-1 causing local vasoconstriction [6-9]. The effect of ET-1 is most prominent in the pulmonary circulation where the ET_A _and ET_B _receptors are widely distributed [10,11]. Previous investigators have noticed that intravenously infused ET-1 results in increased pulmonary artery pressure and lung edema [12,13]. Moreover, in isolated rat lungs in which the vasculature has been paralyzed, ET-1 enhances microvascular permeability, but the mechanisms involved have not yet been settled [14].

Studies *in vitro *have shown that the binding of ET-1 to its receptor might induce the activation of protein kinase C (PKC) [15,16]. Activation of the α isoform of PKC (PKC-α) might cause disturbances in the shape of the cells as well as of the intercellular junctions. The latter changes might promote acute lung injury (ALI) [17-19]. However, we are unable to determine whether any study *in vivo *has tested whether PKC-α is activated in endotoxin-induced ALI.

In sheep subjected to continuous infusion of endotoxin, we recently found that the dual ET_A _and ET_B _receptor blocker tezosentan precludes ALI as evaluated by improved gas exchange and a partial reversal of the increases in pulmonary vascular pressures and extravascular lung water index (EVLWI) [9]. However, the mechanisms involved in the tezosentan-induced reduction of EVLWI still remain obscure. We speculate whether non-selective blockade of ET-1 receptor by tezosentan alleviates ALI by dampening the activation of PKC-α and modulating inflammatory mediators such as tumor necrosis factor-α (TNF-α) and interleukin-8 (IL-8).

The aim of the present study was twofold: first, to investigate in sheep subjected to endotoxin-induced lung injury whether a relationship exists between the plasma concentration of ET-1 and characteristics of ALI such as the increases in lung microvascular pressure and extravascular lung water content, with or without tezosentan; and second, to assess the effects of tezosentan on the activation of PKC-α in lung tissue in parallel with changes in the plasma concentrations of TNF-α and IL-8.

## Methods

The present investigation is based partly on data from a previously published study from our group [9] that was approved by the Norwegian Experimental Animal Board.

In brief, 17 yearling sheep were instrumented with a pulmonary artery thermal dilution catheter introduced via an introducer in the left external jugular vein, a thermo-dye dilution catheter introduced via an introducer in the ipsilateral common carotid artery, and a catheter in the left atrium, as described previously [9].

### Experimental protocol

The animals were randomly assigned to a sham-operated group (*n *= 3), a group (*n *= 7) receiving an intravenous infusion of *Escherichia coli *lipopolysaccharide (LPS) 15 ng/kg per min for 24 hours (LPS group), and a group (*n *= 7) subjected to LPS and, from 4 hours, an intravenous injection of tezosentan 3 mg/kg followed by infusion at 1 mg/kg per hour for the reminder of the 24-hour experiment (LPS plus tezosentan group). During the experiment, sheep had free access to food and water.

EVLWI was assessed by the thermal-dye dilution method (Cold Z-021; Pulsion Medical Systems, Munich, Germany). Pulmonary micro-occlusion pressure (Pmo) was determined every 4 hours, as described previously [20]. In brief, Pmo was determined by advancing the Swan-Ganz catheter into the occlusion position in a distal pulmonary artery with the balloon deflated. The criteria for attainment of the micro-occlusion position included: first, easy retrograde aspiration of blood from the catheter; second, a pH, partial pressure of oxygen (PO_2_) and carbon dioxide (PCO_2_) of aspirated blood consistent with occlusion position, that is, partial pressure of oxygen in occlusion position higher than arterial partial pressure of oxygen (PmoO_2 _> PaO_2_) and partial pressure of carbon dioxide in occlusion position higher than arterial partial pressure of carbon dioxide (PmoCO_2 _< PaCO_2_); third, micro-occlusion pressure greater than proximal occlusion pressure; and fourth, micro-occlusion pressure greater than left atrial pressure, with true zero confirmed by connecting the left atrial catheter and the Swan-Ganz catheter sequentially to the same fixed transducer. Blood for biochemical analysis was sampled at 0, 4, 12, and 24 hours. After the sheep had been killed, lung samples were taken and kept in liquid nitrogen for further analyses.

### Western blotting

The activation of PKC-α was assessed by translocation of kinase from cytosolic and/or membrane fractions of lung tissue extracts. In brief, lung tissue samples were homogenized (Polytron homogenizer, blade rotation speed 5,000 r.p.m.) in 1 ml of ice-cold extraction buffer consisting of (in mmol/l): 250 sucrose, 1 EDTA, 1 EGTA, 20 Tris-HCl pH 7.5, 10 2-mercaptoethanol, 20 dithiothreitol and 1 tablet of Complete™ EDTA-free protease inhibitor cocktail per 10 ml. Crude extracts were centrifuged at 200*g *to remove debris, followed by 100,000*g *for 60 min at 4°C. The supernatant represented the cytosolic fraction. The pellet was resuspended by sonication in 200 ml of a similar buffer supplemented with 1% Triton X-100 and centrifuged at 25,000*g *for 15 min at 4°C. The supernatant was collected as the Triton X-100-soluble membrane fraction. For SDS-PAGE, 10% polyacrylamide gels were loaded with 10 mg of protein per lane. After the end of electrophoresis, proteins were electroblotted to nitrocellulose membranes. Membranes were probed overnight with anti-PKC-α primary antibodies (Santa Cruz Biotechnology, Santa Cruz, CA, USA) at 4°C and for 1 hour with sheep anti-rabbit horseradish peroxidase-conjugated secondary antibodies (Zymed, San Francisco, CA, USA) at 22°C. Blots were incubated with ChemiLucent detection kit (Chemicon, Temecula, CA, USA). Immunopositive bands of PKC-α were detected with a Kodak Image Station 1000 (Kodak, Rochester, NY, USA) and densitometry readings were taken for statistical analysis.

### Biochemical measurements

The ET-1 plasma levels were measured by chemiluminescent enzyme immunoassay (QuantiGlo QET00; R&D Systems, Minneapolis, MN, USA). Plasma levels of TNF-α and of IL-8 were determined with an Immulite instrument (Diagnostic Products Corporation, Los Angeles, CA, USA).

### Statistical analysis

Data were checked for normal distribution by the Kolmogorov–Smirnov test. The relationship between ET-1, Pmo, and EVLWI was evaluated by regression analysis with the Pearson correlation coefficient. Equality of regression lines between the LPS and the LPS plus tezosentan groups was tested by single multiple regression [21]. The detected relative amounts of PKC-α in the groups and tissue fractions were compared by one-way analysis of variance. Plasma concentrations of ET-1, TNF-α, and IL-8 were analyzed by analysis of variance for repeated measurements. If *F *was statistically significant, Scheffe's test was used for *post hoc *intergroup analysis. To evaluate differences within groups towards the baseline value (time 0 hours), we used test of contrasts. We regarded *P *< 0.05 as statistically significant.

## Results

**Figure 1:**
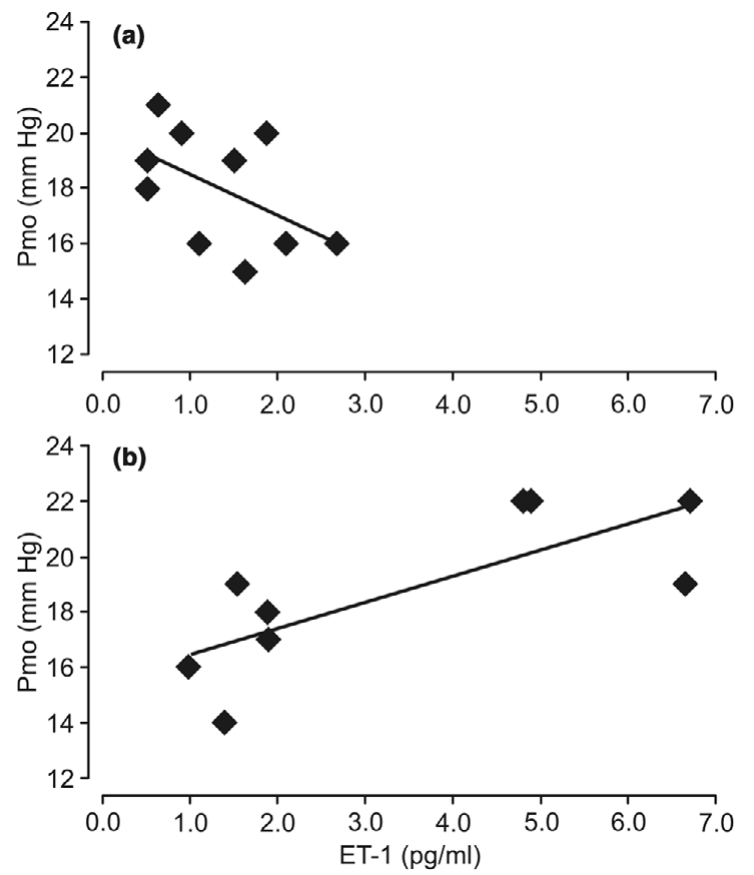
Relationship between plasma concentration of endothelin-1 (ET-1) and pulmonary micro-occlusion pressure (Pmo) in sheep. **(a) **From 0 to 12 hours of LPS infusion (*r *= -0.51, *P *= 0.12, *n *= 10); **(b) **from 12 to 24 hours of LPS infusion (*r *= 0.75, *P *< 0.01, *n *= 9).

**Figure 2:**
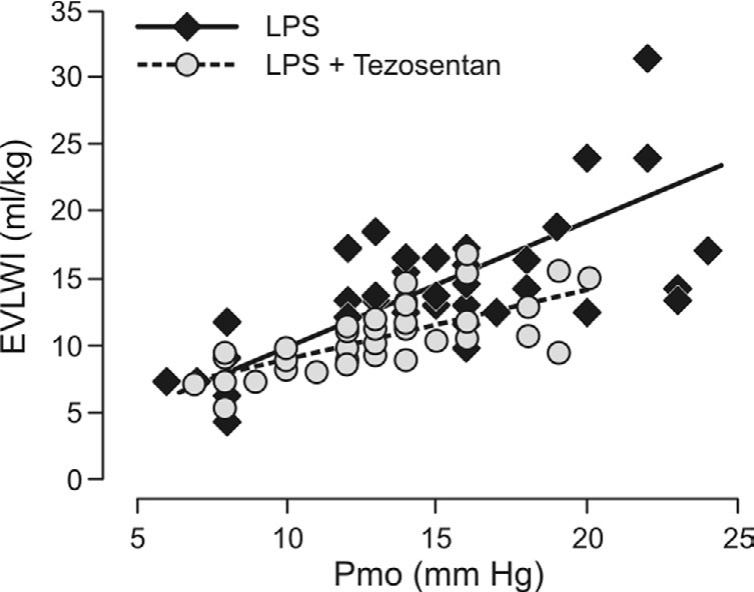
Relationship between extravascular lung water index (EVLWI) and pulmonary micro-occlusion pressure (Pmo) in endotoxemic sheep. LPS alone (r = 0.73, P < 0.01, n = 42). LPS with tezosentan (r = 0.67, P < 0.0001, n = 42).

In sham-operated sheep, all variables remained unchanged throughout the 24-hour experiments. During the first 12 hours we found no significant correlation between the plasma concentration of ET-1 and Pmo in sheep subjected to LPS (Fig. 1a). In contrast, we found a positive correlation between these variables beyond 12 hours (*P *< 0.01; Fig. 1b). A positive correlation also existed between Pmo and EVLWI in the LPS and the LPS plus tezosentan groups, as depicted in Fig. 2. However, tezosentan reduced the slope of the regression line compared with LPS alone (*P *< 0.05; Fig. 2).

**Figure 3:**
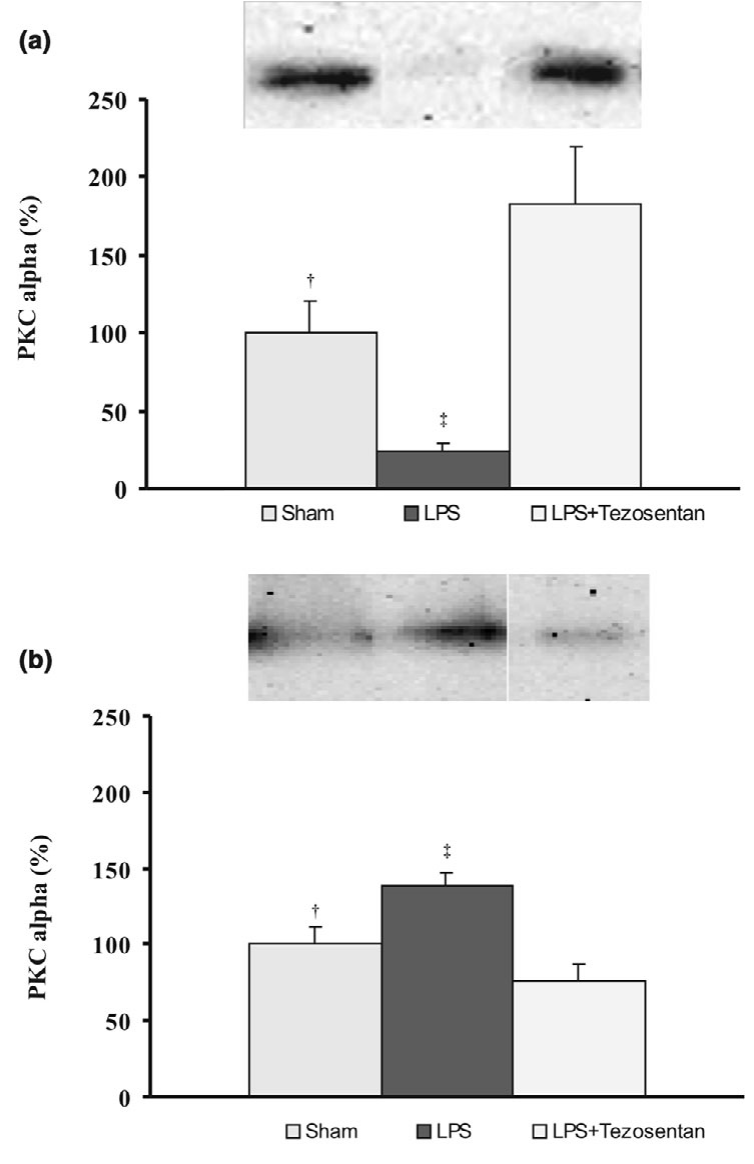
Protein kinase Cα (PKC-α) in sheep lung tissue homogenates detected by Western blotting. **(a) **In the cytosolic fraction; **(b) **in the membrane fraction. Results are means ± SEM. Groups were as follows: sham-operated group (*n *= 3); lipopolysaccharide group (LPS; *n *= 4); LPS plus tezosentan group (*n *= 4). ^†^*P *< 0.05 between sham-operated and LPS groups; ^‡^*P *< 0.05 between LPS and LPS plus tezosentan groups.

As shown in Fig. 3, extracts of lung tissue from sheep exposed to LPS displayed a 75% reduction of the cytosolic fraction of PKC-α in comparison with samples from sham-operated animals (*P *< 0.05). The membrane fraction of PKC-α simultaneously increased by 40% in the LPS group compared with sham-operated sheep. Administration of tezosentan completely prevented the translocation of PKC-α from the cytosolic to the membrane fractions.

**Figure 4:**
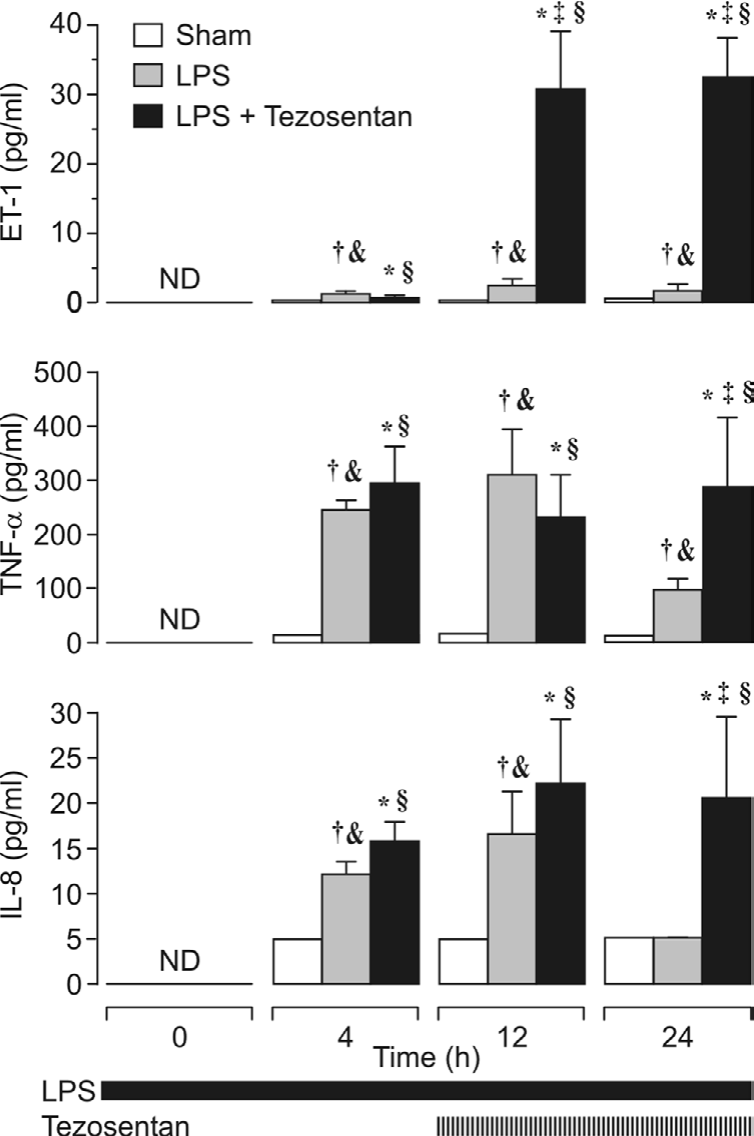
Plasma concentration of endothelin-1 (ET-1), tumor necrosis factor-α (TNF-α) and interleukin-8 (IL-8). Results are means ± SEM. Groups were as follows: sham-operated group (*n *= 3); lipopolysaccharide group (LPS; *n *= 7); LPS plus tezosentan group (*n *= 7). ND, not detectable. ^†^*P *< 0.05 between sham-operated and LPS groups; ^‡^*P *< 0.05 between LPS and LPS plus tezosentan groups; *P < 0.05 between sham-operated and LPS plus tezosentan groups; ^&^*P *< 0.05 from *t *= 0 hours in the LPS group; ^§^*P *< 0.05 from *t *= 0 hours in the tezosentan group.

Figure 4 shows that after 4 hours of exposure to LPS, in parallel with the rise in ET-1, the plasma concentrations of TNF-α and IL-8 increased compared with intragroup baseline and sham-operated animals (*P *< 0.05). Notably, on cessation of the experiment, plasma concentrations of ET-1, TNF-α, and IL-8 were significantly higher in the LPS plus tezosentan group than with LPS alone (*P *< 0.05).

## Discussion

The present study shows that during the late phase of endotoxemia in sheep (12 to 24 hours), the plasma concentration of ET-1 is significantly correlated with the microvascular pressure, whereas no such correlation was found during the early phase. Moreover, we observed a significant and positive correlation throughout the experiment between microvascular pressure and EVLWI in both endotoxemic groups. Interestingly, the regression line had a significantly lower slope in animals receiving tezosentan. To our knowledge, this is the first study demonstrating that blockade of ET-1 receptors precludes endotoxin-induced changes in PKC-α in cytosolic and membrane fractions of lung tissue.

Pulmonary microvascular pressure and permeability are important determinants of lung edema [22]. As reported previously by our group and others, increases in EVLWI in animals exposed to infusion of LPS are associated with enhanced pulmonary microvascular pressure [9,23,24]. However, none of these investigators have focused on the relationship between the plasma concentration of ET-1 and the pulmonary microvascular pressure. There is therefore no general agreement about where in the course of illness, or how, ET-1 exerts its action. One previous report suggests that ET-1 contributes directly to the severity of ALI by increasing the pulmonary microvascular pressure from the first hours of endotoxemia [25]. However, at variance with these results, we found that a fairly strong correlation between the plasma concentration of ET-1 and Pmo exists only in the late phase of endotoxemia. This is also in accordance with investigators who argue that thromboxane A_2 _is the dominating mediator of vasoconstriction during the first hours of endotoxemia, whereas ET-1 is responsible for vasoconstriction in the late phase [26-29].

The significantly positive correlation between Pmo and EVLWI in endotoxemia, and the decrease in the relationship after treatment with tezosentan, agrees fully with a recent investigation in endotoxemic pigs [30]. However, the beneficial effects of ET-1 blockade cannot be explained solely by attenuation of the endotoxin-induced increase in pulmonary artery pressure. The declining slope of the regression line between Pmo and EVLWI in tezosentan-treated animals indicates that additional factors affecting lung fluid filtration might be active. A few years ago, investigators found that ET-1 increases fluid filtration in isolated blood-perfused rat lungs pretreated with papaverine to deprive the lungs of any vascular tone [14]. Because the ability of the lung microvascular pressure to increase was precluded, the authors interpreted their findings as a result of increased permeability. However, the exact mechanisms involved still remain obscure.

PKC consists of a set of different isoenzymes: classical (α, β, γ), novel (ε, δ, θ, η), and atypical (ξ, λ), of which only the classical isoforms are sensitive to changes in intracellular Ca^2+ ^concentration [31]. Recent studies have shown that ET-1 stimulates the release of Ca^2+ ^from the endoplasmic reticulum and activates PKC-α in the cell membrane [15-19]. After being activated, PKC-α has been shown to mediate the disruption of vascular endothelial cadherin junctions [32]. Moreover, PKC-α activates myosin light chain kinase, which is involved in endothelial cell gap formation and barrier dysfunction [33]. In the lung vasculature, PKC-α-induced disruption might derange the endothelial integrity [19]. We therefore speculate that the increase in vascular permeability and the evolution of ALI might be due to ET-1-induced activation of PKC-α in the cell membrane. We believe that blockade of ET-1 receptors, resulting in a combination of reduced microvascular pressure and decreased activation of PKC-α, is one of the main reasons for the amelioration of ALI in the present study.

It is well established that infusion of LPS stimulates a release of inflammatory mediators such as TNF-α, IL-8, and ET-1 [34-36]. In contrast, ET-1 stimulates monocytes and macrophages to release TNF-α and IL-8 in its own right [37,38]. In the present study, enhanced plasma concentrations of TNF-α, IL-8, and ET-1 were found after 4 hours in both endotoxemic groups. However, at the end of the experiments the plasma concentrations of all three mediators were significantly higher in tezosentan-treated animals than in animals given LPS alone. The increases in ET-1 and TNF-α are consistent with a previous investigation employing the endothelin receptor antagonist bosentan [39], but in contrast to that short-term study, we exposed sheep to 24 hours of endotoxemia. According to another recent study, ET_B _receptors in the lungs are involved in the clearance of ET-1 from the circulation [40]. Consequently, ET-1 receptor blockade prolongs ET-1 half-life in the plasma and reportedly shifts tissue uptake from the lungs to other organs [41]. In the present study, tezosentan increased the plasma concentration of ET-1 to an extent that might have enhanced the release of TNF-α and IL-8 from the monocytes and macrophages.

The present endotoxin-induced lung injury model in sheep is not ideal for elucidating the effects of ET-1 receptor blockade on permeability because microvascular pressure cannot be deliberately changed. Further studies of ET-1 receptor blockade on permeability are therefore required in a more complex experimental setting on intact animals or in isolated perfused lungs.

## Conclusion

In endotoxemic sheep, ET-1 plasma concentration is significantly correlated with Pmo in the late phase. Moreover, Pmo and extravascular lung water content demonstrate a positive correlation from the first hours of endotoxin infusion. Blockade of ET-1 receptors attenuates ALI by reducing the pulmonary microvascular pressure and most probably also by decreasing permeability secondary to reducing the activation of PKC-α. However, further studies are needed to explain the exact mechanisms behind the decrease in extravascular lung water and the prevention of activation of PKC-α after ET-1 receptor blockade.

## Key messages

• 	In endotoxemic sheep, extravascular lung water content correlates positively with pulmonary microvascular pressure.

• 	Non-selective endothelin-1 receptor blockade attenuates ovine endotoxin-induced lung injury by reducing pulmonary microvascular pressure and probably also by decreasing microvascular permeability secondary to reduced activation of PKCα.

## Abbreviations

ALI = acute lung injury; ET-1 = endothelin-1; EVLWI = extravascular lung water index; IL = interleukin; LPS = lipopolysaccharide; PKC = protein kinase C; Pmo = pulmonary capillary micro-occlusion pressure; TNF = tumor necrosis factor.

## Competing interests

This study was supported by Helse Nord (Norwegian governmental funds), project number 4001.721.132 and departmental funds of the Departments of Anesthesiology, Physiology and Clinical Chemistry, University of Tromsø, Norway.

## Authors' contributions

VK participated in the design of the study, analyzed the data, and drafted the manuscript. MK, MS, TA, OCI and KY contributed to the biochemical analysis and participated in the design of the study. LB administered the study, participated in the design of the study and suggested improvements to the manuscript. All authors read and approved the final manuscript.

## References

[B1] Yanagisawa M, Kurihara H, Kimura S, Tomobe Y, Kobayashi M, Mitsui Y, Yazaki Y, Goto K, Masaki T (1988). A novel potent vasoconstrictor peptide produced by vascular endothelial cells. Nature.

[B2] Mitaka C, Hirata Y, Nagura T, Tsunoda Y, Amaha K (1993). Circulating endothelin-1 concentrations in acute respiratory failure. Chest.

[B3] Douglas SA, Beck GR, Elliott JD, Ohlstein EH (1995). Pharmacological evidence for the presence of three distinct functional endothelin receptor subtypes in the rabbit lateral saphenous vein. Br J Pharmacol.

[B4] de Nucci G, Thomas R, D'Orleans-Juste P, Antunes E, Walder C, Warner TD, Vane JR (1988). Pressor effects of circulating endothelin are limited by its removal in the pulmonary circulation and by the release of prostacyclin and endothelium-derived relaxing factor. Proc Natl Acad Sci USA.

[B5] Masaki T (1995). Possible role of endothelin in endothelial regulation of vascular tone. Annu Rev Pharmacol Toxicol.

[B6] Pernow J, Hemsen A, Lundberg JM (1989). Increased plasma levels of endothelin-like immunoreactivity during endotoxin administration in the pig. Acta Physiol Scand.

[B7] Nakamura T, Kasai K, Sekiguchi Y, Banba N, Takahashi K, Emoto T, Hattori Y, Shimoda S (1991). Elevation of plasma endothelin concentrations during endotoxin shock in dogs. Eur J Pharmacol.

[B8] Mitaka C, Hirata Y, Makita K, Nagura T, Tsunoda Y, Amaha K (1993). Endothelin-1 and atrial natriuretic peptide in septic shock. Am Heart J.

[B9] Kuklin VN, Kirov MY, Evgenov OV, Sovershaev MA, Sjoberg J, Kirova SS, Bjertnaes LJ (2004). Novel endothelin receptor antagonist attenuates endotoxin-induced lung injury in sheep. Crit Care Med.

[B10] Henry PJ, Rigby PJ, Self GJ, Preuss JM, Goldie RG (1990). Relationship between endothelin-1 binding site densities and constrictor activities in human and animal airway smooth muscle. Br J Pharmacol.

[B11] McKay KO, Black JL, Diment LM, Armour CL (1991). Functional and autoradiographic studies of endothelin-1 and endothelin-2 in human bronchi, pulmonary arteries, and airway parasympathetic ganglia. J Cardiovasc Pharmacol.

[B12] Horgan MJ, Pinheiro JM, Malik AB (1991). Mechanism of endothelin-1-induced pulmonary vasoconstriction. Circ Res.

[B13] Filep JG, Sirois MG, Rousseau A, Fournier A, Sirois P (1991). Effects of endothelin-1 on vascular permeability in the conscious rat: interactions with platelet-activating factor. Br J Pharmacol.

[B14] Helset E, Kjaeve J, Hauge A (1993). Endothelin-1-induced increases in microvascular permeability in isolated, perfused rat lungs requires leukocytes and plasma. Circ Shock.

[B15] Griendling KK, Tsuda T, Alexander RW (1989). Endothelin stimulates diacylglycerol accumulation and activates protein kinase C in cultured vascular smooth muscle cells. J Biol Chem.

[B16] Danthuluri NR, Brock TA (1990). Endothelin receptor-coupling mechanisms in vascular smooth muscle: a role for protein kinase C. J Pharmacol Exp Ther.

[B17] Lynch JJ, Ferro TJ, Blumenstock FA, Brockenauer AM, Malik AB (1990). Increased endothelial albumin permeability mediated by protein kinase C activation. J Clin Invest.

[B18] Siflinger-Birnboim A, Goligorsky MS, Del Vecchio PJ, Malik AB (1992). Activation of protein kinase C pathway contributes to hydrogen peroxide-induced increase in endothelial permeability. Lab Invest.

[B19] Siflinger-Birnboim A, Johnson A (2003). Protein kinase C modulates pulmonary endothelial permeability: a paradigm for acute lung injury. Am J Physiol Lung Cell Mol Physiol.

[B20] Bjertnaes LJ, Koizumi T, Newman JH (1998). Inhaled nitric oxide reduces lung fluid filtration after endotoxin in awake sheep. Am J Respir Crit Care Med.

[B21] Kleinbaum DG, Kupper LL, Muller KE, Kleinbaum DG (1998). Method II: using a single regression equation to compare two straight lines. Applied Regression Analysis and Multivariable Methods.

[B22] Block ER (1992). Pulmonary endothelial cell pathology: implications for acute lung injury. Am J Med Sci.

[B23] Traber DL, Adair TH, Adams T (1981). Hemodynamic consequences of endotoxemia in sheep. Circ Shock.

[B24] Kutzsche S, Lyberg T, Bjertnaes LJ (2001). Effects of adenosine on extravascular lung water content in endotoxemic pigs. Crit Care Med.

[B25] Albertini M, Ciminaghi B, Mazzola S, Clement MG (2001). Improvement of respiratory function by bosentan during endotoxic shock in the pig. Prostaglandins Leukot Essent Fatty Acids.

[B26] Henry C, Ogletree M, Brigham K, Hammon JW (1991). Attenuation of the pulmonary vascular response to endotoxin by a thromboxane synthesis inhibitor (UK-38485) in unanesthetized sheep. J Surg Res.

[B27] Morel DR, Pittet JF, Gunning K, Hemsen A, Lacroix JS, Lundberg JM (1991). Time course of plasma and pulmonary lymph endothelin-like immunoreactivity during sustained endotoxaemia in chronically instrumented sheep. Clin Sci.

[B28] Weitzberg E (1993). Circulatory responses to endothelin-1 and nitric oxide with special reference to endotoxin shock and nitric oxide inhalation. Acta Physiol Scand Suppl.

[B29] Schmeck J, Heller A, Groschler A, Recker A, Neuhof H, Urbaschek R, Koch T (2000). Impact of endothelin-1 in endotoxin-induced pulmonary vascular reactions. Crit Care Med.

[B30] Rossi P, Wanecek M, Konrad D, Oldner A (2004). Tezosentan counteracts endotoxin-induced pulmonary edema and improves gas exchange. Shock.

[B31] Mellor H, Parker PJ (1998). The extended protein kinase C superfamily. Biochem J.

[B32] Sandoval R, Malik AB, Minshall RD, Kouklis P, Ellis CA, Tiruppathi C (2001). Ca^2+ ^signalling and PKCα activate increased endothelial permeability by disassembly of VE-cadherin junctions. J Physiol.

[B33] Garcia JG, Davis HW, Patterson CE (1995). Regulation of endothelial cell gap formation and barrier dysfunction: role of myosin light chain phosphorylation. J Cell Physiol.

[B34] Sugiura M, Inagami T, Kon V (1989). Endotoxin stimulates endothelin-release in vivo and in vitro as determined by radioimmunoassay. Biochem Biophys Res Commun.

[B35] Beutler B, Cerami A (1989). The biology of cachectin/TNF – a primary mediator of the host response. Annu Rev Immunol.

[B36] Mathison JC, Wolfson E, Ulevitch RJ (1988). Participation of tumor necrosis factor in the mediation of gram negative bacterial lipopolysaccharide-induced injury in rabbits. J Clin Invest.

[B37] Helset E, Sildnes T, Seljelid R, Konopski S (1993). Endothelin-1 stimulates human monocytes in vitro to release TNF-1α, IL-1β and IL-6. Mediators Inflamm.

[B38] Helset E, Sildnes T, Konopski S (1994). Endothelin-1 stimulates monocytes in vitro to release chemotactic activity identified as interleukin-8 and monocyte chemotactic protein-1. Mediators Inflamm.

[B39] Wanecek M, Oldner A, Rudehill A, Sollevi A, Alving K, Weitzberg E (1997). Cardiopulmonary dysfunction during porcine endotoxin shock is effectively counteracted by the endothelin receptor antagonist bosentan. Shock.

[B40] Shiba R, Yanagisawa M, Miyauchi T, Ishii Y, Kimura S, Uchiyama Y, Masaki T, Goto K (1989). Elimination of intravenously injected endothelin-1 from the circulation of the rat. J Cardiovasc Pharmacol.

[B41] Burkhardt M, Barton M, Shaw SG (2000). Receptor- and non-receptor-mediated clearance of big-endothelin and endothelin-1: differential effects of acute and chronic ETA receptor blockade. J Hypertens.

